# Macronutrient composition of street food in Central Asia: Bishkek, Kyrgyzstan

**DOI:** 10.1002/fsn3.1753

**Published:** 2020-08-20

**Authors:** Gabriela Albuquerque, Inês Lança de Morais, Marcello Gelormini, Sofia Sousa, Susana Casal, Olívia Pinho, Pedro Moreira, João Breda, Nuno Lunet, Patrícia Padrão

**Affiliations:** ^1^ EPIUnit ‐ Instituto de Saúde Pública Universidade do Porto Porto Portugal; ^2^ Division of Noncommunicable Diseases and Life‐Course World Health Organization (WHO) Regional Office for Europe Copenhagen Denmark; ^3^ Faculdade de Ciências da Nutrição e Alimentação da Universidade do Porto Porto Portugal; ^4^ LAQV/REQUIMTE Laboratório de Bromatologia e Hidrologia Universidade do Porto Porto Portugal; ^5^ Centro de Investigação em Atividade Física Saúde e Lazer Universidade do Porto Porto Portugal; ^6^ WHO European Office for the Prevention and Control of Noncommunicable Diseases WHO Regional Office for Europe Moscow Russian Federation; ^7^ Departamento de Ciências da Saúde Pública e Forenses e Educação Médica Faculdade de Medicina da Universidade do Porto Porto Portugal

**Keywords:** Central Asia, Food Processing, Kyrgyzstan, Nutritional Value, Ready‐Prepared Foods, Street Food

## Abstract

**Background:**

Urban areas in central Asia are currently undergoing nutrition transition. Street food is very popular, but the specific foods available and their nutritional composition are unknown. The aim was to describe the availability and macronutrient composition of street foods in Bishkek, Kyrgyzstan.

**Results:**

Trained interviewers collected data on street food vending sites’ characteristics and food availability (*n* = 596). Samples of the most commonly available foods and drinks were collected (*n* = 80 homemade; *n* = 40 industrial). Macronutrients were quantified through chemical analysis. Fruit, beverages, and food other than fruit were available in 4.0%, 61.7%, and 81.0% of the vending sites, respectively. Among those selling food other than fruit, 56.5% sold only homemade (e.g., bread, main dishes, snacks, pastries, sandwiches, and cakes), 23.3% both homemade and industrial and 20.2% only industrial foods (e.g., bread, snacks, pastries, cakes, and cookies). Homemade foods presented the highest energy/serving (median kcal/serving: 357 versus 145, *p* < .001). A high content in saturated and trans‐fatty acids was observed in some homemade traditional dishes and snacks, reaching, respectively, 30.2 g/serving and 2.9 g/serving (in homemade *manty,* a traditional dish). Tea and soft drinks were available in over 50% of the vending sites selling beverages.

**Conclusion:**

The high availability of street food in Bishkek highlights its importance for this urban population. Traditional snacks, dishes, and beverages coexist with more westernized products. The variability in energy, macronutrients, and lipid profile of homemade and industrial products reflects heterogeneous culinary practices and ingredients. Policies promoting the availability of healthy foods and ingredients should be implemented.

## Graphical abstract

Street food vending sites were selected in public markets in Bishkek (Kyrgyzstan), through random and systematic procedures; vendors (*n* = 596) were interviewed and samples (*n* = 120) of the most commonly available foods and beverages were collected. Mostly homemade, but also industrial street foods, including traditional and westernized options, were highly frequent. Several street foods presented high saturated and trans‐fat content.

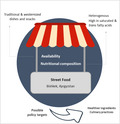

## 1 INTRODUCTION

Low‐ and middle‐income countries (LMIC) in central Asia are currently facing changes in eating habits, associated with economic development (Food & Agriculture Organization, 2017; Kelly, 2016). This phenomenon known by nutrition transition is generally characterized by an increase in food availability and consumption (Popkin, 1999; Popkin & Gordon‐Larsen, 2004), along with more time spent away from home and eating out more frequently (Popkin, 1999; Winarno & Allain, 1991). This is reflected into declines in the consumption of nonprocessed or minimally processed foods, which are nutritionally more dense (e.g., fruit, vegetables, legumes, and whole grains) and increases in the consumption of processed and ultraprocessed foods, more likely to be energy dense, rich in fat, particularly saturated (SFA) and trans‐fatty acids (TFA), sugar and salt (Popkin, 1999).

Along with these rapid changes in eating habits and lifestyles are the increasing burden of diet‐related noncommunicable diseases (NCDs) (Popkin & Gordon‐Larsen, 2004). A high burden of NCDs has been described in central Asia (Food & Agriculture Organization, 2017). In Kyrgyzstan, cardiovascular diseases are the leading cause of death, accounting for 53% of all deaths in 2016 (World Health Organization, 2018) and the prevalence of overweight and obesity is increasing, both in adults and children (World Health Organization, 2015), despite undernutrition persists as a relevant health concern in the country (World Health Organization, 2015).

In contexts of low resources and working long distances from home, such as in urban areas of central Asia, street food plays an important role on the diet of many consumers: it is cheap and easily accessible (Liu, Zhang, & Zhang, 2014; Steyn et al., 2014; Winarno & Allain, 1991) and frequently replaces home meals (Steyn et al., 2014),(Bhowmik, 2005). Only a few studies have focused on the nutritional value of street food (Abrahale, Sousa, Albuquerque, Padrao, & Lunet, 2019). Although there is a great heterogeneity regarding its nutritional composition, depending on the ingredients used, preparation and food processing methods (Draper, 1996; Namugumya & Muyanja, 2012; Steyn et al., 2014), it has been estimated that street food largely contributes to the total intakes of fat, particularly TFA, salt and sugar (Steyn et al., 2014).

There is currently a lack of representative data about dietary habits of urban populations in central Asia, and specifically, in Kyrgyzstan (Rippin et al., 2018). The objective of this study was to characterize the street food environment in Bishkek, Kyrgyzstan, focusing on the vending places and their food availability, and to describe the macronutrient composition of the most commonly available homemade and industrial foods and beverages.

## 2 METHODS

### 2.1 Setting and study design

This study was implemented within the scope of the FEEDcities project, supported by the World Health Organization (WHO)—Europe, which used a stepwise standardized methodology to characterize the street food environment in countries in Central Asia and Eastern Europe (World Health Organization, 2019). The definition of street food adopted in this study was the one proposed by the Food and Agriculture Organization and the WHO, as “ready‐to‐eat foods and beverages prepared and/or sold by vendors or hawkers especially in the streets and other similar places” (Food & Agriculture Organization, 1989; World Health Organization, 1996).

This cross‐sectional evaluation was specifically designed to assess the availability and nutritional composition of the street food in Bishkek, capital city of Kyrgyzstan and was conducted between June and July 2016. Kyrgyzstan is a lower‐middle‐income country, characterized by a slight economic growth in recent years, of which one of the main supports is the agriculture sector. The country has about six million people (Central Inteligency Agency, 2019; World Bank, 2019). Bishkek is the main urban area and largest city in the country, with 996 000 inhabitants, approximately 17% of the national population (Central Inteligency Agency, 2019).

In a site visit conducted for the planning of data collection, members of the research team observed that most street food vendors in Bishkek were concentrated within or in the proximity of public markets. Thus, from a list of 19 public markets identified by local authorities, a fixed proportion (approximately 50%) was randomly chosen, resulting in 10 markets. The study areas were delimited by 500‐meter diameter buffers around each selected market (with the centroid in its geographic midpoint), covering the market and its surroundings.

Eligible vending sites were defined as the business establishments selling ready‐to‐eat food, including beverages and/or snacks, from any venue other than permanent storefront businesses or establishments with four permanent walls not selling directly to the street, operating in the predefined perimeter. This included mobile vendors, as well as sellers with semi‐static or stationary vending units. The exclusion criteria were the following: (a) food establishments with four permanent walls; (b) permanent storefront businesses; (c) street vendors selling exclusively nonfood products or raw foods not ready‐to‐eat; (d) food stalls and carts which were part of permanent stores or licensed establishments.

### 2.2 Data collection: vending sites, vendors, and food availability

The markets were assessed on consecutive days, during 3 weeks, ensuring the representation of both weekdays and weekends. Ten interviewers, operating in pairs, canvassed the study areas in search of street food vending sites, starting by the assessment of the whole market and then moving to the surroundings. After registering the Global Positioning System (GPS) coordinates of each vending site, they collected the following information, through direct observation: sex of the vendor, mobility of the vending site, and type of physical setup of stationary vending sites. Stationary vending sites were classified into formal (stand, dukoni; table with chairs for customers or truck) or informal (freezers, soft ice‐cream vending machines, bench with table board, push cart, and tandoor).

Afterward, the interviewers systematically invited vendors from one in every two vending sites (based on an initially estimated mean number of 100 eligible vending sites per market) to participate in the study. They explained the study objectives and procedures and asked for an express consent to participate in the study. When the vendor agreed, they carried out computer‐assisted personal interview, enquiring about vending site ownership, characteristics (access to drinking water, to toilet facilities and electricity), food vending activity (operating periods—during the week; during the year and under which weather conditions) and food availability, including products sold and serving sizes. A shortened version, ensuring a brief description of each of these categories, was conducted with the food vendors operating mobile vending sites, given their limited time to answer the questionnaire.

A total of 1,216 eligible street food vending sites were identified and 604 vendors were invited to participate, of whom 596 (98.7%) accepted. No statistically significant differences were found between participant and nonparticipant vendors regarding mobility and the physical setup of the stationary vending sites.

Foods available was grouped according to their nature, into fruit (product *in natura*, either fresh or dry), beverages (any alcoholic and nonalcoholic beverage), or food other than fruit. Food other than fruit was further classified as homemade (foods of domestic manufacture cooked and/or prepared at home or on the street, even if using industrial ingredients) or industrial (food products produced by food industry and sold as is without further preparation and/or cooking, encompassing both processed and unprocessed foods (Monteiro et al., 2018)). Homemade food was also grouped in “cooked,” “prepared but uncooked,” or “uncooked and unprepared”. Beverages were further classified into water, soft drinks, fruit juice‐based drinks, fresh fruit juice, energy drinks, milk, milk‐based drinks, coffee, tea, traditional beverages, or alcoholic beverages.

### 2.3 Food sample collection and nutritional composition assessment

Samples of the most commonly available foods and beverages (hereafter referred to as “food,” although beverages were included) of unknown composition (identified previously through the data on food availability) were collected for nutritional composition assessment. The most frequent homemade foods (*n* = 20) were *piroshky*, *samsa*, bread, *kurut*, *kompot*, bun, *belyashi*, sandwich, *manty*, *keksi*, *chebureki*, hamburger, boiled corn, prepared salads (namely carrot salad), *ashlyamfu*, cake, hot‐dog, *lagman*, sausage roll, and porridge. The most frequent industrial foods (*n* = 10) were sunflower seeds *in natura*, cookies, chewing gum, bun, chocolate, chips, croutons, lollypop, wafer, and candies, but given that the nutritional composition of sunflower seeds *in natura*, chewing gums, lollypop, and candies is not expected to vary significantly, the following most available industrial foods were selected instead: *chalap*, *keksi*, *maksym* (local beverage), and corn snacks. Four samples of each of these items were collected from different vending sites, in a total of 120, corresponding to 80 homemade and 40 industrial.

The selection of the vending sites where the food samples were collected was based on the list of eligible vending sites previously assessed. In each day, the GPS coordinates of 10 vending sites in a predefined market were randomly selected. One sample of homemade and one sample of industrial food, corresponding to one serving, were bought whenever possible at these vending sites. If not possible, a systematic selection procedure was followed, in which field researchers started moving north from that point and change direction clockwise (first east, then south, then west, then north again) whenever the limits of the study area or a physical barrier (such as a wall or a canal) were met, until reaching vending sites where these foods were available (World Health Organization, 2019). The 120 food samples were collected in 10 consecutive days.

After collection, samples were homogenized, weighted, and stored in a freezer (−18ºC) until the nutritional composition assessment (World Health Organization, 2019), which included the analysis of a) moisture, by oven drying at 103°C until constant weight; b) protein, by Kjedahl's method; c) fat, by Soxhlet's method and d) fatty acids [SFA, monounsaturated (MUFA), polyunsaturated (PUFA), n‐3 and n‐6 fatty acids, TFA], by gas chromatography for the fatty acid methyl esters (Cruz et al., 2013). Total carbohydrates plus fiber were estimated by difference (Horwitz, 2000). The energy value of samples was estimated after proximate analysis of food components (moisture, protein, total fat, and ash), performed in accordance with standard methods, as recommended by the Association of Official Analytical Chemists (AOAC) (Horwitz, 2000). The analytical results were the average of two determinations per food sample. A third determination was conducted only when the first two were not in agreement and, in this case, the average of the two concordant results was calculated. All the analytical results were expressed by serving size, in grams (g).

### 2.4 Statistical Analyses

Absolute and relative frequencies (categorical variables) were used to describe the vending sites and food availability. Pearson's chi‐square test was used to compare food availability and vending sites’ characteristics. Markets were defined as the sampling units. The statistical analyses were conducted adjusting for the clustering at the sampling unit level.

Regarding the nutritional composition assessment, given that more than one sample was obtained for each food, in different vending sites, mean serving sizes per food, in grams (g), were calculated as the mean weight of the individual samples collected. The presented content of each nutrient in selected foods was obtained as a mean of the content in the individual samples. Per‐serving levels of each nutrient were expressed in (g)/ serving. Results were presented for each food, as the mean and range of energy (Kcal) and macronutrients (g) per serving. The nonparametric Mann–Whitney U test was used for comparing the energy content of homemade and industrial foods. A critical level of significance (p) <0.05 was considered statistically significant.

Statistical analyses were performed using the software STATA® version 11.0 (StataCorp., College Station, Texas, USA).

### 2.5 Ethical considerations

This study was conducted according to the guidelines laid down in the Declaration of Helsinki and all procedures involving human subjects were approved by the Ethics Committee of the Institute of Public Health of the University of Porto (CE16058). Verbal informed consent was obtained from all vendors.

## 3 RESULTS

**Figure 1:**
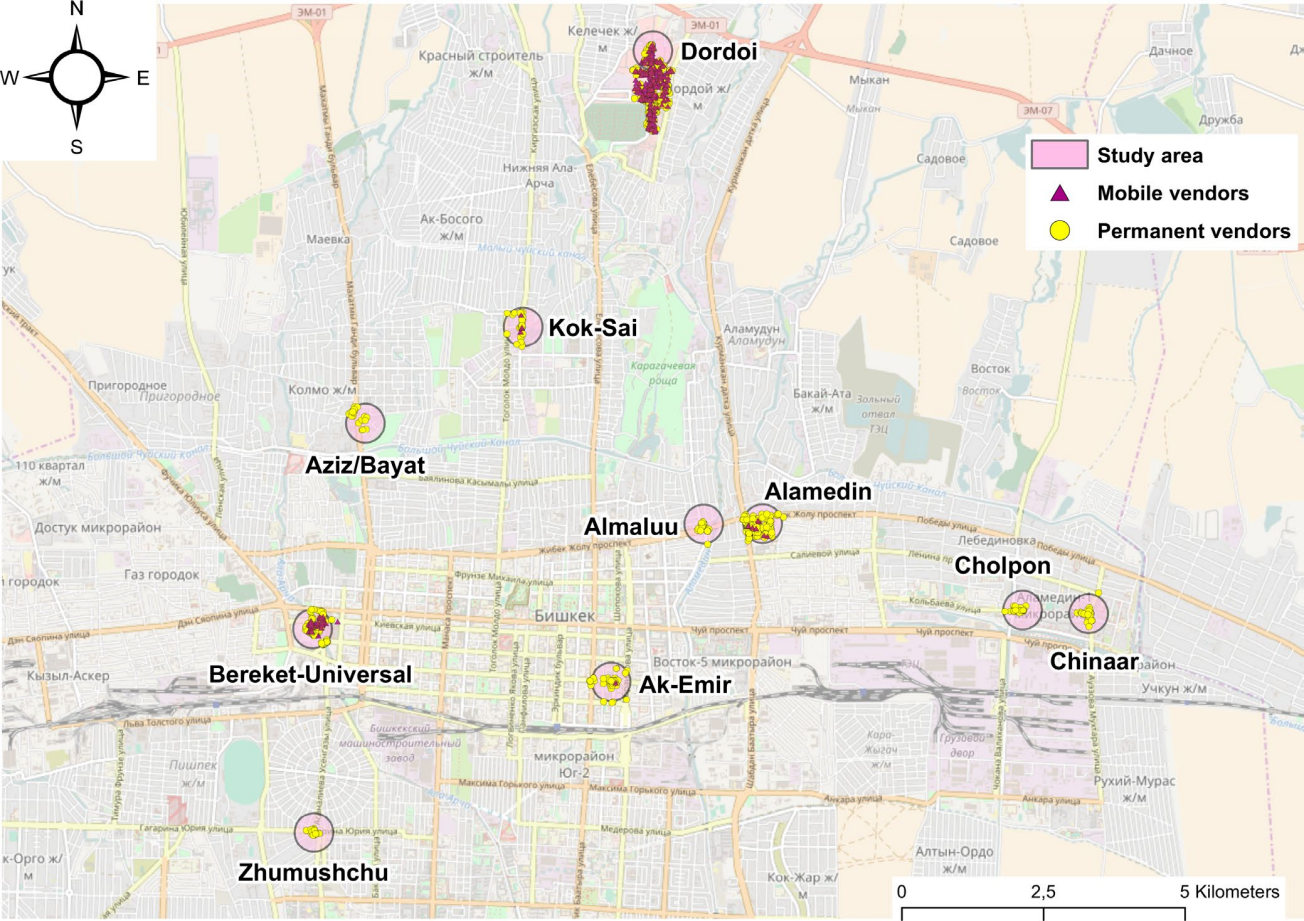
Geographical distribution of the markets and street food vending sites in Bishkek, Kyrgyzstan

Figure 1 depicts the markets selected for this study, scattered throughout the city of Bishkek, and the vending sites evaluated. Within each market, the distribution of the vending sites ranged from predominantly concentrated in specific market sections, as in the *Almalulu*, *Ak‐Emir*, *Cholpon*, and *Zhumushchu* markets, to scattered across wider areas, as in the *Alamedin*, *Aziz*/*Bayat*, *Bereket*‐*Universal*, *Chinaar*, *Dordoi,* and *Kok‐Sai* markets.

### 3.1 Food vendors and vending sites

Nearly four in every five vendors found in Bishkek were women (78.2%) and were working on a stationary vending site (72.7%). Most vendors from mobile vending sites were owners of their business (88.3% versus 42.0%, *p* = .001). Access to toilet facility (97.1%) and drinking water (95.5%) was available for most vending sites (Table 1), the latter mostly among stationary formal physical setups (98.9% versus 94.8%, *p* = .044). Stationary vending sites were mostly operating all year long (71.4%), seven days a week (82.7%), and under every type of weather (74.4%). The informal stationary vending sites were less likely to work the whole year (51.7% versus 76.5%, *p* < .001) or under every type of weather (58.4% versus 78.5%, *p* < .001) (Table S1).

**Table 1:** Characteristics of street food vending sites and food availability, by type of vending site in Bishkek, Kyrgyzstan (*n* = 596)

	Total (*n* = 596)		Stationary (*n* = 433)		Mobile (*n* = 163)		*p*
	*n*	%	*n*	%	*n*	%	
Vending sites characteristics							
Food Vendor sex (women)	466	78.2	332	76.7	134	82.2	.009
Food vendor ownership	326	54.7	182	42.0	144	88.3	.001
Access to drinking water	569	95.5	414	95.6	155	95.1	.813
Access to toilet facility	579	97.1	418	96.5	161	98.8	.215
**Food availability**							
Fruit^a^	24	4.0	19	4.4	5	3.1	0.153
Food other than fruit	483	81.0	336	77.6	147	90.2	0.001
Industrial^b^	97	20.2	78	23.2	19	13.2	0.394
Homemade and Industrial^b^	112	23.3	78	23.2	34	23.6	
Homemade^b^	271	56.5	180	53.6	91	63.2	
Cooked^c^	310	80.9	199	77.1	111	88.8	0.001
Prepared but noncooked^c^	117	30.5	75	29.1	42	33.6	0.519
Nonprepared and noncooked^c^	3	0.8	2	0.8	1	0.8	0.979
Beverages^d^	367	61.7	269	62.3	98	60.1	0.804
Tea	210	57.2	129	48.0	81	82.7	0.006
Soft drinks	205	55.9	183	68.0	22	22.4	<0.001
Water	158	43.1	146	54.3	12	12.2	<0.001
Coffee	123	33.5	48	17.8	75	76.5	0.197
Fruit juice‐based drink	121	33	103	38.3	18	18.4	<0.001
Traditional beverages^e^	94	25.6	60	22.3	34	34.7	<0.001
Alcoholic beverages^f^	40	10.9	39	14.5	1	1.0	<0.001
Energy drinks	13	3.5	13	4.8	0	0.0	0.390
Milk	8	2.2	7	2.6	1	1.0	0.027
Fresh fruit juice	3	0.8	3	1.1	0	0.0	0.277
Milk‐based drinks^g^	2	0.5	2	0.7	0	0.0	0.219

^a^ Sample size for this variable is lower, due to missing data (*n* = 594)

^b^ Sample size for this variable is lower, due to missing data (*n* = 480)

^c^ The sum of the values for this variable is higher than the total number of homemade foods, as each vendor could offer different ways of preparing foods.

^d^ Sample size for this variable is lower, due to missing data (*n* = 595)

^e^ Traditional beverages: k*ompot* or *m*ors (*n* = 50), m*aksym* (*n* = 44), *chalap* (*n* = 36), *ayran* (*n* = 11), *jarma* (*n* = 9), yoghurt (*n* = 5), kephyr (*n* = 2), aralash (*n* = 1), sherbet (*n* = 1), and tams*han* (*n* = 1)

^f^ Alcoholic beverages: cocktail (*n* = 21), *kvass* (*n* = 12), beer (*n* = 8), *kymyz* (*n* = 7), vodka (*n* = 5), *bozo* (*n* = 5), wine (*n* = 2), and cognac (*n* = 1)

^g^ Milk‐based drinks: milk drinks and cocktails (*n* = 1) and cocoa (*n* = 1)

### 3.2 Food availability

Fruit, beverages, and food other than fruit were sold in 4.0%, 61.7%, and 81.0% of the vending sites, respectively. Food other than fruit was significantly more available in mobile food vending sites (90.2 versus 77.6%, *p* = .001). Overall, most vending places sold only homemade food (56.5%), followed by those that sold both homemade and industrial (23.3%), and only industrial foods (20.2%). The mobile vending sites sold frequently more homemade foods, while the stationary vending sites sold industrial foods more often. Cooked homemade street foods were more frequently available in mobile (88.8% versus 77.1%, *p* = .001) vending sites (Table 1). Among stationary vending sites, the formal ones also sold more often cooked homemade dishes (80.3% versus 65.1%, *p* = .007), whereas prepared noncooked homemade foods were more frequently available in informal vending sites (41.9% versus 26.8%, *p* = .003) (Table S1).

Overall, no statistically significant differences were found regarding the availability of beverages between mobile and stationary vending sites (Table 1) nor between formal and informal stationary vending sites, except for coffee which was more frequently sold in formal physical setups (Table S1). Tea was the most frequently available beverage, found in 57.2% of the vending sites selling beverages, mostly mobile, followed by soft drinks (55.9%) and water (43.1%), mostly in stationary vending sites (Table 1).

### 3.3 Nutritional composition

Industrial foods were more energy dense than homemade foods (median kcal/100g: 433 versus 239, *p* < .001), but the homemade foods presented higher energy content per serving (median kcal/ serving: 357 versus 145, *p* < .001). The mean energy values per serving were higher in homemade traditional dishes such as *manty* (968 kcal/serving) and *lagman* (739 kcal/serving). The latter was also the food with highest content in carbohydrates (109.3 g/serving) followed by cake (103.9 g/serving). Homemade traditional dishes and snacks showed the highest mean protein [*manty* (38.9 g/serving); *lagman* (29.7 g/serving)]; and total fat [*manty* (51.4 g/serving; *samsa* (28.8 g/serving)] content per serving (Table 2).

**Table 2:** Nutritional composition per serving (energy and macronutrients) of the street food samples from Bishkek, Kyrgyzstan, evaluated by chemical analysis

	**Energy and macronutrients**																		
	***N***	**Mean serving size (min–max)** **(g/serving)**			**Mean energy (min–max)** **(kcal/serving)**			**Mean protein (min–max)** **(g/serving)**			**Mean carbohydrates (min–max)** **(g/serving)**			**Mean total fat (min–max) (g/serving)**			**Mean water (min–max) (g/serving**		
Industrial food																			
Bun	4	45	(23	‐ 63)	150	(84	‐ 198)	3.8	(0.7	‐ 6.1)	30.1	(16.7	‐ 39.5)	1.5	(0.8	‐ 2.4)	9.7	(4.1	‐ 17.4)
*Chalap*	4	200	(200	‐ 200)	39	(26	‐ 63)	4.3	(2.5	‐ 6.2)	2.8	(1.3	‐ 5.1)	1.1	(0.3	‐ 2.9)	189.6	(185.7	‐ 191.7)
Chips	4	23	(20	‐ 25)	129	(114	‐ 146)	1.4	(1.2	‐ 1.6)	11.5	(10.1	‐ 12.1)	8.6	(7.6	‐ 10.2)	0.3	(0.3	‐ 0.4)
Chocolate	4	42	(36	‐ 49)	211	(187	‐ 256)	2.4	(1.3	‐ 3.7)	28.2	(22.4	‐ 35.6)	9.8	(4.4	‐ 13.8)	1.0	(0.5	‐ 1.8)
Cookies	4	34	(29	‐ 45)	160	(139	‐ 199)	2.5	(1.8	‐ 4.1)	22.7	(19.3	‐ 29.6)	6.6	(5.0	‐ 7.9)	1.8	(0.7	‐ 3.3)
Corn snacks	4	38	(38	‐ 38)	170	(162	‐ 181)	5.4	(1.7	‐ 16.3)	24.7	(12.8	‐ 29.6)	5.5	(4.1	‐ 7.1)	2.1	(1.6	‐ 2.7)
Croutons	4	34	(30	‐ 36)	139	(123	‐ 147)	4.1	(3.5	‐ 4.5)	25.7	(22.6	‐ 27.0)	2.2	(2.0	‐ 2.3)	0.9	(0.8	‐ 0.9)
*Maksym*	4	200	(200	‐ 200)	56	(38	‐ 68)	2.5	(1.6	‐ 3.8)	10.1	(6.1	‐ 12.8)	0.7	(0.5	‐ 0.9)	185.2	(182.3	‐ 189.6)
Sweet pastries	4	34	(23	‐ 41)	138	(109	‐ 182)	1.9	(1.7	‐ 2.4)	20.5	(11.7	‐ 26.9)	5.4	(2.7	‐ 7.4)	5.4	(3.5	‐ 7.8)
Wafers	4	83	(32	‐ 107)	428	(174	‐ 547)	4.2	(2.3	‐ 5.7)	55.8	(18.2	‐ 73.5)	20.9	(10.2	‐ 25.9)	1.8	(0.8	‐ 2.5)
**Homemade food**																			
*Ashlyamfu*	4	520	(350	‐ 825)	451	(367	‐ 609)	23.4	(13.3	‐ 33.3)	71.8	(37.7	‐ 95.7)	7.7	(5.1	‐ 11.3)	411.1	(254.8	‐ 676.3)
*Belyashi*	4	162	(148	‐ 181)	424	(325	‐ 523)	13.5	(11.6	‐ 15.2)	56.8	(50.2	‐ 65.1)	15.9	(5.5	‐ 22.5)	72.1	(61.1	‐ 83.2)
Bread	4	120	(120	‐ 120)	342	(321	‐ 377)	13.0	(12.4	‐ 14.0)	70.3	(66.7	‐ 73.9)	1.0	(0.3	‐ 2.8)	33.3	(26.3	‐ 38.0)
Cake	4	170	(122	‐ 240)	636	(393	‐ 982)	11.3	(7.5	‐ 17.4)	103.9	(63.7	‐ 162.1)	19.5	(12.0	‐ 29.4)	33.5	(28.9	‐ 37.2)
*Chebureki*	4	135	(128	‐ 143)	408	(397	‐ 415)	12.3	(11.8	‐ 12.5)	50.5	(48.2	‐ 53.7)	17.4	(16.1	‐ 19.1)	52.8	(46.9	‐ 62.3)
Corn (boiled)	4	278	(205	‐ 349)	285	(233	‐ 370)	10.6	(8.4	‐ 13.2)	48.8	(40.9	‐ 68.0)	5.3	(3.8	‐ 7.4)	209.5	(148.8	‐ 261.5)
Hamburger	4	245	(161	‐ 331)	571	(406	‐ 751)	23.2	(15.9	‐ 30.6)	72.5	(48.0	‐ 110.5)	20.9	(16.7	‐ 25.0)	123.8	(77.9	‐ 162.8)
Hot dog	4	283	(233	‐ 363)	618	(524	‐ 821)	23.7	(19.9	‐ 31.4)	80.3	(62.0	‐ 111.0)	22.5	(15.1	‐ 27.9)	150.5	(123.4	‐ 184.7)
*Keksi*	4	75	(48	‐ 100)	282	(194	‐ 362)	5.8	(3.2	‐ 8.4)	40.4	(25.9	‐ 58.0)	10.8	(8.7	‐ 14.7)	16.7	(8.1	‐ 26.8)
*Kompot*	4	200	(200	‐ 200)	68	(39	‐ 93.)	0.3	(0.2	‐ 0.5)	16.3	(9.5	‐ 22.2)	0.2	(0.1	‐ 0.4)	182.9	(176.9	‐ 190.1)
*Kurut*	4	23	(16	‐ 27)	49	(35	‐ 55)	8.6	(6.3	‐ 10.1)	2.4	(1.7	‐ 2.9)	0.6	(0.3	‐ 0.9)	8.7	(5.2	‐ 10.1)
*Lagman*	4	603	(562	‐ 650)	739	(677	‐ 858)	29.7	(20.7	‐ 39.8)	109.3	(105.3	‐ 111.9)	20.3	(11.9	‐ 28.9)	436.5	(408.7	‐ 463.0)
*Manty*	4	430	(398	‐ 508)	968	(796	‐ 1,344)	38.9	(28.6	‐ 44.8)	87.5	(71.6	‐ 117.4)	51.4	(39.4	‐ 78.2)	245.2	(233.1	‐ 260.7)
*Piroshky*	4	106	(67	‐ 134)	293	(225	‐ 364)	6.7	(4.5	‐ 8.4)	42.6	(30.1	‐ 54.5)	10.6	(7.3	‐ 13.7)	44.2	(20.9	‐ 57.7)
Porridge	4	249	(196	‐ 313)	254	(202	‐ 346)	7.7	(4.6	‐ 9.3)	48.6	(27.2	‐ 71.1)	3.2	(0.9	‐ 6.4)	187.1	(151.8	‐ 247.2)
Salad (carrot)	4	212	(160	‐ 286)	189	(138	‐ 286)	4.8	(1.8	‐ 10.0)	21.8	(16.0	‐ 37.3)	9.2	(6.3	‐ 13.1)	170.5	(128.6	‐ 221.1)
*Samsa*	4	176	(86	‐ 250)	560	(288	‐ 750)	20.6	(6.6	‐ 33.3)	54.7	(28.4	‐ 72.9)	28.8	(16.5	‐ 39.5)	68.1	(33.8	‐ 105.2)
Sandwich	4	138	(85	‐ 190)	395	(253	‐ 524)	14.6	(11.0	‐ 17.1)	44.7	(30.2	‐ 58.2)	17.5	(9.8	‐ 24.8)	58.4	(31.8	‐ 86.0)
Sausage roll	4	103	(76	‐ 129)	286	(202	‐ 339)	10.0	(7.6	‐ 13.2)	39.3	(23.4	‐ 60.1)	9.8	(5.0	‐ 13.5)	41.2	(34.6	‐ 48.0)
Sweet bun	4	66	(50	‐ 83)	215	(183	‐ 253)	6.5	(5.2	‐ 8.6)	39.5	(31.9	‐ 48.2)	3.4	(1.6	‐ 5.4)	16.1	(7.9	‐ 22.8)

Regarding the lipid profile, homemade dishes and snacks presented the highest content in SFA (*manty*: 30.2 g/serving; *samsa*: 12.4 g/serving), MUFA (*manty*: 15.1 g/serving; *samsa*: 9.3 g/serving) and in PUFA (*lagman*: 11.4 g/serving; hot dog: 9.4 g/serving). Particularly, the highest content in n‐6 fatty acids was found in homemade *lagman* (11.2 g/serving), *belyashi* (9.1 g/serving), and hot‐dog (9.1 g/serving). N‐3 fatty acids were only present in reduced amounts in most foods, reaching 0.5 g/serving in homemade *manty*. The highest content in TFA was found in industrial wafers (3.8 g/serving), but also in homemade traditional dishes such as *manty* (2.9 g/serving) (Table 3).

**Table 3:** Nutritional composition per serving (fatty acid profile) of the street food samples from Bishkek, Kyrgyzstan, evaluated by chemical analysis

		**Fatty acid profile**																				
	***N***	**Mean serving size (min–max)** **(g/serving)**			**Mean SFA (min–max)** **(g/serving)**			**Mean MUFA (min–max)** **(g/serving)**			**Mean PUFA (min–max)** **(g/serving)**			**Mean *n*‐ 6 (min–max)** **(g/serving)**			**Mean *n*‐ 3 (min–max)** **(g/serving)**			**Mean TFA (min–max)** **(g/serving)**		
Industrial food																						
Bun	4	45	(23	‐ 63)	0.4	(0.2	‐ 0.6)	0.5	(0.2	‐ 0.8)	0.5	(0.3	‐ 1.1)	0.5	(0.3	‐ 1.1)	0.0	(0.0	‐ 0.0)	0.1	(0.0	−0.2)
*Chalap*	4	200	(200	‐ 200)	0.7	(0.2	‐ 1.8)	0.3	(0.1	‐ 0.8)	0.0	(0.0	‐ 0.1)	0.0	(0.0	‐ 0.1)	0.0	(0.0	‐ 0.0)	0.1	(0.0	−0.2)
Chips	4	23	(20	‐ 25)	1.0	(0.9	‐ 1.2)	1.7	(1.5	‐ 1.9)	5.9	(5.2	‐ 7.1)	5.9	(5.2	‐ 7.0)	0.0	(0.0	‐ 0.0)	0.0	(0.0	−0.0)
Chocolate	4	42	(36	‐ 49)	4.6	(2.6	‐ 7.2)	3.8	(1.4	‐ 5.1)	1.0	(0.4	‐ 1.5)	0.9	(0.3	‐ 1.4)	0.0	(0.0	‐ 0.0)	0.5	(0.1	−1.2)
Cookies	4	34	(29	‐ 45)	2.7	(2.2	‐ 3.4)	2.3	(1.9	‐ 2.7)	0.9	(0.6	‐ 1.5)	0.9	(0.5	‐ 1.4)	0.0	(0.0	‐ 0.0)	0.6	(0.0	−1.1)
Corn snacks	4	38	(38	‐ 38)	1.5	(0.7	‐ 2.9)	2.2	(0.8	‐ 3.6)	1.7	(0.6	‐ 2.8)	1.6	(0.6	‐ 2.8)	0.1	(0.0	‐ 0.5)	0.0	(0.0	−0.1)
Croutons	4	34	(30	‐ 36)	0.3	(0.2	‐ 0.4)	0.6	(0.5	‐ 0.7)	1.2	(1.2	‐ 1.3)	1.2	(1.2	‐ 1.3)	0.0	(0.0	‐ 0.0)	0.0	(0.0	−0.0)
*Maksym*	4	200	(200	‐ 200)	0.2	(0.1	‐ 0.5)	0.2	(0.1	‐ 0.2)	0.2	(0.1	‐ 0.3)	0.2	(0.1	‐ 0.3)	0.0	(0.0	‐ 0.0)	0.0	(0.0	−0.1)
Sweet pastries	4	34	(23	‐ 41)	3.2	(1.3	‐ 5.7)	1.3	(1.0	‐ 1.7)	0.7	(0.4	‐ 1.3)	0.6	(0.3	‐ 1.3)	0.0	(0.0	‐ 0.0)	0.2	(0.0	−0.3)
Wafers	4	83	(32	‐ 107)	9.8	(6.2	‐ 15.1)	6.1	(2.8	‐ 9.6)	1.2	(0.5	‐ 2.7)	1.1	(0.5	‐ 2.6)	0.0	(0.0	‐ 0.0)	3.8	(0.3	−8.3)
**Homemade food**																						
*Ashlyamfu*	4	520	(350	‐ 825)	1.5	(0.9	‐ 2.7)	2.1	(1.1	‐ 3.7)	4.0	(2.9	‐ 5.5)	3.9	(2.8	‐ 5.4)	0.1	(0.1	‐ 0.2)	0.1	(0.0	−0.2)
*Belyashi*	4	162	(148	‐ 181)	2.5	(1.5	‐ 3.4)	3.8	(1.3	‐ 6.4)	9.2	(2.5	‐ 13.9)	9.1	(2.5	‐ 13.8)	0.0	(0.0	‐ 0.1)	0.3	(0.2	−0.4)
Bread	4	120	(120	‐ 120)	0.3	(0.1	‐ 1.1)	0.3	(0.1	‐ 1.1)	0.3	(0.2	‐ 0.6)	0.3	(0.2	‐ 0.5)	0.0	(0.0	‐ 0.0)	0.0	(0.0	−0.0)
Cake	4	170	(122	‐ 240)	11.3	(8.5	‐ 14.2)	4.5	(1.5	‐ 9.2)	2.9	(1.9	‐ 4.9)	2.8	(1.7	‐ 4.8)	0.1	(0.1	‐ 0.1)	0.1	(0.0	−0.3)
*Chebureki*	4	135	(128	‐ 143)	4.2	(3.3	‐ 5.7)	4.4	(4.0	‐ 5.0)	8.4	(6.8	‐ 10.0)	8.3	(6.7	‐ 9.9)	0.1	(0.1	‐ 0.1)	0.8	(0.0	−1.3)
Corn (boiled)	4	278	(205	‐ 349)	1.1	(0.6	‐ 1.5)	1.9	(1.5	‐ 2.6)	2.2	(1.7	‐ 3.2)	2.2	(1.7	‐ 3.0)	0.1	(0.0	‐ 0.2)	0.4	(0.3	−0.6)
Hamburger	4	245	(161	‐ 331)	6.5	(3.9	‐ 10.0)	5.9	(4.4	‐ 7.3)	7.8	(6.5	‐ 8.6)	7.6	(6.3	‐ 8.4)	0.1	(0.1	‐ 0.2)	0.0	(0.0	−0.0)
Hot dog	4	283	(233	‐ 363)	5.1	(2.7	‐ 6.1)	7.6	(4.1	‐ 9.2)	9.4	(7.7	‐ 12.5)	9.1	(7.6	‐ 12.2)	0.2	(0.1	‐ 0.3)	0.7	(0.4	−1.2)
*Keksi*	4	75	(48	‐ 100)	2.0	(1.4	‐ 3.3)	2.6	(2.1	‐ 3.2)	5.9	(2.2	‐ 9.3)	5.8	(2.1	‐ 9.2)	0.1	(0.0	‐ 0.1)	0.4	(0.3	−0.6)
*Kompot*	4	200	(200	‐ 200)	0.1	(0.0	‐ 0.2)	0.1	(0.0	‐ 0.1)	0.0	(0.0	‐ 0.1)	0.0	(0.0	‐ 0.1)	0.0	(0.0	‐ 0.0)	0.2	(0.1	−0.4)
*Kurut*	4	23	(16	‐ 27)	0.4	(0.2	‐ 0.6)	0.1	(0.1	‐ 0.2)	0.0	(0.0	‐ 0.0)	0.0	(0.0	‐ 0.0)	0.0	(0.0	‐ 0.0)	0.0	(0.0	−0.0)
*Lagman*	4	603	(562	‐ 650)	3.6	(1.8	‐ 6.2)	4.8	(2.3	‐ 8.3)	11.4	(7.7	‐ 13.2)	11.2	(7.5	‐ 12.9)	0.1	(0.1	‐ 0.2)	0.1	(0.0	−0.1)
*Manty*	4	430	(398	‐ 508)	30.2	(23.7	‐ 48.7)	15.1	(10.2	‐ 21.0)	3.3	(2.5	‐ 4.4)	2.7	(2.1	‐ 3.8)	0.5	(0.3	‐ 0.6)	0.5	(0.1	−1.3)
*Piroshky*	4	106	(67	‐ 134)	1.4	(1.3	‐ 1.8)	2.2	(1.6	‐ 2.8)	6.7	(4.3	‐ 8.7)	6.6	(4.2	‐ 8.6)	0.0	(0.0	‐ 0.0)	2.9	(1.6	−4.1)
Porridge	4	249	(196	‐ 313)	1.6	(0.5	‐ 2.9)	1.0	(0.3	‐ 2.3)	0.5	(0.1	‐ 1.0)	0.4	(0.1	‐ 0.9)	0.0	(0.0	‐ 0.1)	0.2	(0.1	−0.4)
Salad (carrot)	4	212	(160	‐ 286)	1.1	(0.6	‐ 1.6)	2.0	(1.6	‐ 2.4)	6.0	(3.4	‐ 9.0)	6.0	(3.4	‐ 8.9)	0.0	(0.0	‐ 0.1)	0.1	(0.1	−0.2)
*Samsa*	4	176	(86	‐ 250)	12.4	(6.2	‐ 21.2)	9.3	(5.7	‐ 12.0)	5.6	(4.1	‐ 7.2)	5.1	(3.9	‐ 7.0)	0.4	(0.1	‐ 1.2)	0.1	(0.0	−0.1)
Sandwich	4	138	(85	‐ 190)	4.3	(3.5	‐ 5.4)	5.5	(3.3	‐ 7.5)	7.2	(2.8	‐ 11.9)	7.0	(2.7	‐ 11.5)	0.2	(0.1	‐ 0.4)	1.6	(0.5	−2.3)
Sausage roll	4	103	(76	‐ 129)	2.1	(1.2	‐ 2.7)	3.1	(1.7	‐ 4.1)	4.5	(2.1	‐ 6.6)	4.4	(2.0	‐ 6.4)	0.1	(0.1	‐ 0.2)	0.4	(0.1	−0.5)
Sweet bun	4	66	(50	‐ 83)	0.9	(0.6	‐ 1.0)	1.0	(0.6	‐ 1.3)	1.4	(0.4	‐ 3.0)	1.4	(0.4	‐ 3.0)	0.0	(0.0	‐ 0.0)	0.1	(0.1	−0.2)

Note

SFA, saturated fatty acids. MUFA, monounsaturated fatty acids. PUFA, polyunsaturated fatty acids. TFA, trans‐fatty acids.

## 4 DISCUSSION

The availability of street food in Bishkek is wide and diverse, including a variety of traditional and westernized foods and beverages. A high variability in energy content, macronutrient, and lipid profile values was found among the most commonly available foods in this city, which may reflect heterogeneous ingredients and culinary practices.

The most frequently available street foods other than fruit were homemade and cooked, including a variety of traditional snacks, pastries and main dishes and a great share of more westernized foods (e.g., hamburger, hot‐dog, and sausage roll). Homemade and industrial beverages, the latter including traditional and westernized options such as soft drinks, were also frequently found. This substantial offer of industrialized goods, resulting from greater access to new industrial foods and ingredients, formerly absent from the diet of this population is in line with recent observations from FAO, that Kyrgyzstan is transitioning from the stage 3 “Receding Famine,” to stage 4 “Degenerative Disease” of the Nutrition transition (Food & Agriculture Organization, 2017). This stage is associated with a shift in lifestyles, namely an acculturation of a “western diet,” rich in sweets, fats, and animal‐source foods and low in fruit, vegetables, legumes, and whole grains (Kelly, 2016; Popkin & Gordon‐Larsen, 2004).

Of the 30 street foods whose nutritional composition was analyzed in this study, the homemade foods had larger servings sizes and also energy content per serving, resembling the results found in Dushanbe, Tajikistan, a neighboring country (Albuquerque et al., 2019). A single serving of some of the homemade dishes accounted for a large share of energy, almost half of the daily recommendation for an average adult (e.g., *manty* and *lagman*), and macronutrients. The largest protein content per serving was found, in fact, in homemade traditional dishes, made mostly of stewed meat, either as dough filling (*manty*), or served with noodles (such as *lagman* or *ashlyamfu*), and in fast‐foods sandwiches, such as hamburger and hot‐dog. Only a few of these foods may include vegetables or plant sources of protein, thus one can infer that most of this protein content is of animal origin. Similar findings have been reported in Mozambique, where stewed dishes containing meat or fish were the main sources of this nutrient (Sousa et al., 2018). Altogether such findings reinforce that street food in urban Bishkek may have a central role in feeding citizens throughout the day and that it may highly contribute to the daily protein and energy intake of the population (Steyn et al., 2014).

The main contributors to the high energy content found in the analyzed street foods were carbohydrates and fat, as expected (Steyn et al., 2014). The foods with higher content in carbohydrates per serving were bread, main dishes, and fast food sandwiches such as hamburger or hot dog. A considerable amount of carbohydrates was also present in sweet snacks, both industrial (e.g., bun, sweet pastries, and wafers) and homemade (e.g., bun and cake). This could be due to a high sugar content, usually present in these foods (Becquey & Martin‐Prevel, 2010; Steyn et al., 2014). The high availability of soft drinks adds up to these findings, as they have been identified to be the main source of added sugar in the diet of urban populations from other LMIC (Malik, Willett, & Hu, 2009; Mozaffarian, Hao, Rimm, Willett, & Hu, 2011). It is known that the consumption of soft drinks is on the rise in upper‐middle and LMIC, with countries in central Asia still showing moderate consumption levels (Singh et al., 2015), but expected to increase if no countermeasures are taken. Although it was not possible to discriminate the content in free sugars in the analyzed foods, it is visible that the availability of sugary foods such as chocolates, cookies, and other sweet foods is high in *Bishkek*.

Furthermore, a high fat content was found in homemade steamed and fried dishes and snacks such as *manty* and *samsa* and industrial sweet snacks such as chocolate and wafers, but also in vegetable preparations. In carrot salad, the high fat content was due, mostly, to PUFA and specifically n‐6 fatty acids, suggesting the addition of vegetable oils. The homemade steamed and fried/meat‐based dishes and snacks showed a high fat content, mainly due to SFA and TFA. A similar lipid profile was observed in snacks sold in urban and rural slums in India (Gupta, Downs, Ghosh‐Jerath, Lock, & Singh, 2016), where the oils used to cook were sources of these fatty acids. Despite insipient research on cooking practices of preparation of street foods, another possible explanation is the fact that although most oils and fats supplied in the retail market may conform with current quality standards, unsafe cooking practices such as continuous reuse of oil for frying may lead to increased TFA concentrations (Esfarjani et al., 2019; Tavakoli, Naderi, Jafari, & Naeli, 2019). Besides, despite the availability of healthier options such as interesterified blends, shortenings or margarine, rich in SFA and TFA, are still traditionally used in bakery cooking given their desirable tenderness, texture and mouthfeel and extending of product shelf life (Farajzadeh Alan, Naeli, Naderi, Jafari, & Tavakoli, 2019). In this sense, in the present work, the heterogeneity of the nutritional composition and energy content found within each food bought in different vending sites may reflect differences in the ingredients used, namely oils and fats, and in the culinary practices, as already described in the literature (Draper, 1996; Namugumya & Muyanja, 2012; Steyn et al., 2014). As such, it seems to be of utmost relevance to study the culinary practices and nutritional literacy of street food vendors and further promote healthier cooking techniques. The study of customers’ motivations for buying street food and their nutritional literacy would be an additional benefit to attain the improvement of the nutritional quality of street foods, but also their acceptance by the population.

Also, even though the vending sites selling exclusively fruit were excluded from our analysis, the fruit and vegetables’ availability in the study sample was low. A study conducted in the country has shown that the daily consumption of fruits and vegetables was positively associated with the number of establishments selling them (Goryakin, Rocco, Suhrcke, Roberts, & McKee, 2015). Other studies have found that increasing the availability of fruits and vegetables in street food vending sites, even when other products are available, was successful in increasing its consumption (Tester, Yen, & Laraia, 2010, 2012). A feasible solution in urban *Bishkek* could be the attribution of economic incentives to the sellers willing to have such healthy products available at their vending sites.

The present study suggests the existence of a window of opportunity for governmental action, toward the improvement of street food nutritional quality. Promoting the higher availability of legumes, fruit, and vegetables and the use of healthier ingredients, specifically fats, while preserving gastronomic identity and culinary practices, would contribute to increase food security in this setting and counteract the effects of nutrition transition. Formal regulations of nutritional content and food labeling or, for example, the introduction of competitive prices for healthier ingredients, both at the food industry and at the small‐scale production level (Downs, Thow, & Leeder, 2013; Hawkes, Jewell, & Allen, 2013) would be possible measures to tackle with the present findings. As part of the membership to the Eurasian Economic Commission, Kyrgyzstan has already taken an important step by implementing a limit of 2g TFA/100g fat in fat products used in the manufacture or preparation of foods (Eurasian Economic Commission, 2011), thus affecting all foods in the market, including street food. The findings of this study highlight the need to invest in monitoring actions targeting both industry and small‐scale production levels given that, out of the 30 analyzed street foods, only three industrial (chips, croutons, and corn snacks) and six homemade (boiled corn, carrot salad, sausage roll, *ashlyamfu*, bread, and *keksi*) complied with this recommendation, in addition to the fact that in foods such as industrial wafer, the TFA content achieved as much as 35 g/100 g of fat.

This exploratory description of the street food environment in Bishkek highlighted its importance for this urban population in central Asia, given its wide expression throughout the city. The stepwise systematic approach for data collection and analysis aimed to provide an unbiased and comprehensive characterization, overcoming additional gaps identified in previous literature (Abrahale et al., 2019; Steyn et al., 2014). Although some results may not be generalized to other communities, given culture‐specific characteristics of the local food environment, the methodology has a wide potential to be adapted to different settings (World Health Organization, 2019), allowing comparison of results. The assessment of the nutritional composition of the most commonly available street foods was based on reliable chemical methods, also overcoming limitations of previous studies (Abrahale et al., 2019; Steyn et al., 2014). Nevertheless, these findings reflect availability rather than consumption and future insight on street food consumption in this setting would strength these assumptions.

## 5 CONCLUSION

Energy‐dense traditional and westernized dishes and snacks, rich in saturated and trans fat were highly available in Bishkek, while healthy food options, such as fruit and vegetables were unfrequently available. Policies to prevent NCDs in Kyrgyzstan should consider the promotion of healthier street foods and the use of healthier ingredients and fats. The involvement of the food industry and street food vendors is essential to guarantee this change in food availability and preserve local traditions and gastronomy.

## 6 DISCLAIMER

João Breda is staff member of the World Health Organization Regional Office for Europe. The author is responsible for the views expressed in this publication, which do not necessarily represent the decisions or stated policy of WHO.

## CONFLICT OF INTEREST

The authors declare that they have no conflict of interest.

## AUTHOR CONTRIBUTION

MG, PM, JB, NL, and PP designed the study. IM and MG supervised the study implementation and data collection. OP and SC were responsible for the chemical analysis of the food samples collected. GA, NL, and PP performed the data analysis and interpretation of the results. GA drafted the manuscript. All authors critically revised the manuscript and gave their final approval of the manuscript submitted for publication.

## Supporting information

Table S1
