# Supplementary material for: Macronutrient composition of street food in Central Asia: Bishkek, Kyrgyzstan
Source: Food Sci Nutr. 2020 Aug 20;8(10):5309–20. doi: 10.1002/fsn3.1753 (PMC7590326; doi:10.1002/fsn3.1753)
Supplement: Supplementary file 1 — Table S1 [file FSN3-8-5309-s001.docx]

| Supplementary Table 1. Characteristics of the stationary street food vending sites and food availability, by type of physical setup in *Bishkek*, Kyrgyzstan (n=433). | | | | | | | |
| --- | --- | --- | --- | --- | --- | --- | --- |
|  | **Total** | | **Physical setup^a^** | | | |  |
|  |  |  | **Informal (n=89)** | | **Formal (n=344)** | | **p** |
| **Food vendor characteristics** | n | **%** | n | **%** | n | **%** |  |
| Food Vendor sex (women) | 332 | 76.7 | 71 | 79.8 | 261 | 75.9 | 0.448 |
| Food vendor ownership | 182 | 42.0 | 40 | 44.9 | 142 | 41.3 | 0.594 |
| **Business characteristics** |  |  |  |  |  |  |  |
| Operating the whole week | 358 | 82.7 | 77 | 86.5 | 281 | 81.7 | 0.105 |
| Operating the whole year | 309 | 71.4 | 46 | 51.7 | 263 | 76.5 | <0.001 |
| Operating under every type of weather | 322 | 74.4 | 52 | 58.4 | 270 | 78.5 | <0.001 |
| **Hygiene and Food Safety** |  |  |  |  |  |  |  |
| Access to electricity | 316 | 73.0 | 54 | 60.7 | 262 | 76.2 | 0.020 |
| Access to drinking water | 414 | 95.6 | 88 | 98.9 | 326 | 94.8 | 0.044 |
| Access to toilet facility | 418 | 96.5 | 87 | 97.8 | 331 | 96.2 | 0.177 |
| **Food Availability** |  |  |  |  |  |  |  |
| **Fruit** | 19 | 4.4 | 7 | 7.9 | 12 | 3.5 | 0.082 |
| **Food other than fruit** | 335 | 77.4 | 53 | 59.6 | 282 | 82.0 | <0.001 |
| Industrial | 79 | 23.6 | 10 | 18.9 | 69 | 24.5 | 0.348 |
| **Homemade and Industrial** | 77 | 23.0 | 10 | 18.9 | 67 | 23.8 |  |
| **Homemade** | 179 | 53.4 | 33 | 62.3 | 146 | 51.8 |  |
| Cooked ^b^ | 199 | 77.7 | 28 | 65.1 | 171 | 80.3 | 0.007 |
| Prepared but not-cooked ^b^ | 75 | 29.3 | 18 | 41.9 | 57 | 26.8 | 0.003 |
| Non-prepared and non-cooked ^b^ | 2 | 0.8 | 1 | 2.3 | 1 | 0.5 | 0.268 |
| **Beverages ^c^** | 269 | 62.3 | 51 | 57.3 | 218 | 63.6 | 0.245 |
| Soft drinks | 183 | 68.0 | 37 | 72.6 | 146 | 67.0 | 0.065 |
| Water | 146 | 54.3 | 28 | 54.9 | 118 | 54.1 | 0.958 |
| Tea | 129 | 48.0 | 26 | 51.0 | 103 | 47.3 | 0.609 |
| Fruit juice-based drink | 103 | 38.3 | 20 | 39.2 | 83 | 38.1 | 0.827 |
| Traditional beverages ^d^ | 60 | 22.3 | 5 | 9.8 | 55 | 25.2 | 0.134 |
| Coffee | 48 | 17.8 | 2 | 3.9 | 46 | 21.1 | 0.003 |
| Alcoholic beverages ^e^ | 39 | 14.5 | 6 | 11.8 | 33 | 15.1 | 0.379 |
| Energy drinks | 13 | 4.8 | 0 | 0.0 | 13 | 6.0 | 0.353 |
| Milk | 7 | 2.6 | 0 | 0.0 | 7 | 3.2 | 0.149 |
| Fresh fruit juice | 3 | 1.1 | 0 | 0.0 | 3 | 1.4 | 0.413 |
| Milk-based drinks ^f^ | 2 | 0.8 | 1 | 2.0 | 1 | 0.5 | 0.346 |
| ^a^ **Informal physical setups:** freezer, soft ice-cream or lemonade machine (n=38)**;** bench with table board (n= 32); push cart (n=16); and *tandoor* (n=3). **Formal physical setups**: stand, stall or booth (n=151); *dukoni* (n=101); table with chairs for customers (n=83); and truck (n=9). | | | | | | | |
| ^b^ The sum of the values for this variable is higher than the total number of homemade foods, as each vendor could offer different ways of preparing foods. | | | | | | | |
| ^c^ For this variable, sample size is lower (n=432). | | | | | | | |
| ^d^ Traditional beverages: *ayran* (n=9), k*ompot* or *m*ors (n=21), m*aksym* (n=39), *chalap* (n=33), *jarma* (n=5), aralash (n=1), sherbet (n=1), tams*han* (n=1), kephyr (n=2) and yoghurt (n=5). | | | | | | | |
| ^e^ Alcoholic beverages: beer (n= 8), wine (n=2), cognac (n=1), vodka (n=5), cocktail (n=21), *bozo* (n=5), *kymyz* (n=6) and *kvass* (n=12). | | | | | | | |
| ^f^ Milk-based drinks: milk drinks and cocktails (n=1) and cocoa (n=1). | | | | | | | |
